# A serotonergic biobehavioral signature differentiates cocaine use disorder participants administered mirtazapine

**DOI:** 10.1038/s41398-022-01934-w

**Published:** 2022-05-06

**Authors:** Liangsuo Ma, Kathryn A. Cunningham, Noelle C. Anastasio, James M. Bjork, Brian A. Taylor, Albert J. Arias, Brien P. Riley, Andrew D. Snyder, F. Gerard Moeller

**Affiliations:** 1 Institute for Drug and Alcohol Studies, Virginia Commonwealth University, Richmond, VA United States; 2 Department of Psychiatry, Virginia Commonwealth University, Richmond, VA United States; 3 Center for Addiction Research and Department of Pharmacology and Toxicology, University of Texas Medical Branch, Galveston, TX United States; 4 Department of Biomedical Engineering, Virginia Commonwealth University, Richmond, VA United States; 5 Virginia Institute for Psychiatric and Behavioral Genetics, Virginia Commonwealth University, Richmond, VA United States

**Keywords:** Addiction, Epigenetics and behaviour

## Abstract

Cocaine use disorder (CUD) patients display heterogenous symptoms and unforeseeable responses to available treatment approaches, highlighting the need to identify objective, accessible biobehavioral signatures to predict clinical trial success in this population. In the present experiments, we employed a task-based behavioral and pharmacogenetic-fMRI approach to address this gap. Craving, an intense desire to take cocaine, can be evoked by exposure to cocaine-associated stimuli which can trigger relapse during attempted recovery. Attentional bias towards cocaine-associated words is linked to enhanced effective connectivity (EC) from the anterior cingulate cortex (ACC) to hippocampus in CUD participants, an observation which was replicated in a new cohort of participants in the present studies. Serotonin regulates attentional bias to cocaine and the serotonergic antagonist mirtazapine decreased activated EC associated with attentional bias, with greater effectiveness in those CUD participants carrying the wild-type 5-HT_2C_R gene relative to a 5-HT_2C_R single nucleotide polymorphism (rs6318). These data suggest that the wild-type 5-HT_2C_R is necessary for the efficacy of mirtazapine to decrease activated EC in CUD participants and that mirtazapine may serve as an abstinence enhancer to mitigate brain substrates of craving in response to cocaine-associated stimuli in participants with this pharmacogenetic descriptor. These results are distinctive in outlining a richer “fingerprint” of the complex neurocircuitry, behavior and pharmacogenetics profile of CUD participants which may provide insight into success of future medications development projects.

## Introduction

Combined cognitive and pharmacotherapeutic approaches optimize recovery from opioid use disorder [1], however, efforts to validate medication candidates for cocaine use disorder (CUD) have largely been negative. Participants are typically screened for inclusion in clinical trials based upon the diagnosis of CUD employing the *Diagnostic and Statistical Manual (DSM) of Mental Disorders* (DSM) [2, 3]. Recent consumption of cocaine is detected by its metabolite benzoylecgonine in urine [4], and the number of clean urine days is a common endpoint for success in clinical trials of CUD therapeutics [5]. However, the diagnosis and detection of cocaine do not encapsulate the profound heterogeneity of individual patients which arises due to developmental and genetic distinctions and interrelated CUD-related pathologies [6–9]. These factors likely contribute to the poor signal detection in clinical trials for CUD medications, and the field recognizes the need to identify and tailor new strategies for maximizing the success of treatment to suppress relapse and extend recovery [10, 11]. One concept is to validate a “biobehavioral signature” which reflects indicators of underlying neurobiological processes and pathogenesis to predict therapeutic success [12, 13]. For example, several genetic markers have been identified as predicting depression, an observation which has recently been validated within an independent cohort of participants with clinically severe depression [14, 15]. Similarly, beyond DSM criteria and identification of a drug-positive urine, readily accessible and objective indicators of CUD processes will allow clinically informed distinctions between CUD patients with the goal to align treatment options to biobehavioral profiles.

Exposure to cocaine-associated cues contributes to continued cocaine use as well as relapse during abstinence and recovery [6, 16, 17]. Our understanding of brain responses to these cues has been greatly enriched by studies employing functional imaging technologies. Notably, fMRI studies have illustrated that CUD impacts not only the function of single brain regions of interest, but importantly the interactions between brain regions which drive behavioral features of the disorder [6]. For example, we recently discovered that the amplified deployment of attentional bias toward cocaine-related stimuli in CUD participants associates with increased anterior cingulate cortex (ACC) to hippocampus effective (directional) connectivity (EC) [18], an observation replicated in opioid use disorder participants [19]. In the present study, this cue-associated ACC→ hippocampus EC activation was selected as a dependent variable to test hypotheses related to the proposed involvement of the serotonergic system in cue-activated brain mechanisms in CUD participants.

Preclinical observations support a prominent role for the serotonin (5-HT) 5-HT_2A_ receptor (5-HT_2A_R) and 5-HT_2C_R systems in the neurobiology of relapse vulnerability [17, 20]. Long-term cocaine self-administration is associated with increased cortical 5-HT_2A_R availability in monkeys [21], suggesting that the sensitivity to cocaine-associated cues during abstinence may be related to the degree of higher 5-HT_2A_R expression (and function) consequent to cocaine self-administration. Notably, selective 5-HT_2A_R antagonists (e.g., M100907, pimavanserin) [22–35] and the 5-HT_2A/2C_R antagonist mirtazapine [26–29] exhibit potency and efficacy to suppress cue-evoked cocaine-seeking in animals and decrease craving, cocaine use, and some psychiatric symptoms in CUD participants [30]. Relatedly, a cortical 5-HT_2C_R knockdown elevated cocaine-seeking and increased local 5-HT_2A_R expression [36, 37], suggesting that the 5-HT_2C_R system is an important regulator of endogenous 5-HT_2A_R control of the neural bases of cocaine cue-evoked behaviors.

The single nucleotide polymorphism (SNP) in the 5-HT_2C_R gene (rs6318) results in hypofunctional cellular signaling in vitro [38, 39]. This SNP also predicts the highest attentional bias toward cocaine-associated stimuli in CUD participants [40]. Given preclinical observations, we postulated that mirtazapine would exhibit greater effectiveness to suppress attentional bias-linked ACC → hippocampus EC in CUD participants with the wild-type *HTR2C* relative to the hypofunctional *HTR2C* SNP. The outcomes confirm a behavioral and pharmacogenetic-fMRI signature of CUD participants which may ultimately aid in maximizing therapeutic success in this persistent disorder.

## Methods

### Participants

This study was approved by the local ethics committee and performed in accordance with the Code of Ethics of the World Medical Association (Declaration of Helsinki). Twenty-eight, non-treatment seeking participants who met DSM-IV criteria for CUD were included in the study (Table 1). Each was genotyped (Taqman Assays-on-Demand, ThermoFisher Scientific, Foster City, CA) for the *HTR2C* gene using an automated allele scoring platform [41], as previously described [40]. Participants were blinded and randomized to receive placebo and mirtazapine (15 mg) two hours prior to the first and second scans, respectively; scans were scheduled approximately two weeks apart. Each participant provided written informed consent, was negative for physical and medical histories as well as psychiatric disorders assessed by the Structured Clinical Interview for DSM-IV [42]. Immediately prior to MRI scanning, each participant was verified to be negative for breath alcohol, and urinalysis for cocaine and other abusable stimulants, barbiturates, benzodiazepines, opioids, Δ [9]-tetrahydrocannabinol, tricyclic antidepressants, and pregnancy (for females). Eight subjects were excluded from the 36 original participants (10 females) who initiated the experiment (see Supplementary Table 1). The remaining 28 participants (*n* = 15 wild-type *HTR2C* and *n* = 13 *HTR2C* SNP) were included for final analysis. Each participant was financially compensated for their time.

**Table 1:** Demographics, substance use parameters and in-scanner behavior under placebo and mirtazapine conditions.

	All (*n* = 28)	Wild-Type *HTR2C* (*n* = 15)	*HTR2C*SNP (*n* = 13)	Statistics
Demographics				
Age (years; mean ± S.D.) Range (years)	44.3 ± 8.6 27–59	46.5 ± 8.5 27–59	41.7 ± 8.3 32–58	*t*_26_ = 1.5, *p* = 0.14
Ethnicity (AA, African American; C, Caucasian)	27 AA, 1 C	14 AA, 1 C	13 AA, 0 C	*p* = 1
Sex (F, female; M, male)	23 M, 5 F	11 M, 4 F	12 M, 1 F	*p* = 0.33
Handedness (AMBI, ambidextrous; L, left; R, right)	2 AMBI 27 R	0 AMBI 15 R	2 AMBI 11 R	*p* = 0.21
Education (years; mean±S.D.) Range (years)	12.3 ± 2.6 7–18	12.2 ± 2.1 7–16	12.5 ± 3.2 7–18	*t*_26_ = 0.3, *p* = 0.77
Substance Use Parameters (mean ± S.D.)				
Lifetime cocaine use (years)	13.9 ± 7.6	13.1 ± 7.0	14.8 ± 8.4	*t*_26_ = 0.58, *p* = 0.56
Cocaine use prior 30 days (days)	13.0 ± 9.3	12.7 ± 10.0	13.3 ± 8.8	*t*_26_ = 0.17, *p* = 0.87
Cocaine administration route	22 smoked 6 nasal	13 smoked 2 nasal	9 smoked 4 nasal	p = 0.37
Lifetime alcohol use (kg)	209.6 ± 286.9	232.9 ± 264.1	182.7 ± 320.0	*t*_26_ = 0.45, *p* = 0.65
Lifetime cigarette use (years)	22.8 ± 12.8	23.2 ± 14.4	22.2 ± 11.1	*t*_26_ = 0.20, *p* = 0.084
Number of cigarettes/day	11.0 ± 8.3	12.5 ± 9.6	9.3 ± 6.4	*t*_26_ = 0.58, *p* = 0.56
Interval between Placebo and Mirtazapine Scans				
Absolute mirtazapine – placebo interval (days; mean±S.D.)	13.86 ± 10.8	17.0 ± 13.6	10.2 ± 4.6	*t*_26_ = 1.70 *p* = 0.10

Student’s *t* test and Fisher’s exact test (two-tailed) were used to test difference between values for participants expressing the wild-type *HTR2C* or the *HTR2C* SNP for the continuous and categorical variables, respectively.

### Cocaine-word Stroop task

We employed the cocaine-word Stroop task [18] within the fMRI scanner to assess attentional bias towards cocaine-related stimuli (see Supplementary Information for details). As in our previous studies [18], attentional bias to cocaine cues was calculated as the mean reaction time (RT) during the cocaine word (CW) blocks minus the mean RT during the neutral word (NW) blocks, i.e., ΔRT = RT (CW) minus RT (NW). Only correct-response trials were included when calculating the mean reaction times.

### fMRI data acquisition

MRI scans were acquired using a Philips Medical Systems (Best, Netherlands) Ingenia wide-bore dStream 3 T MRI scanner, with a 32-channel receive head coil. A spin-echo echo planar pulse sequence was used with: parallel imaging acceleration-factor 2.0, repetition-time 2500 ms, echo-time 75 ms, flip-angle 90 degrees, field-of-view 240 mm (anterior to posterior) × 240 mm (left to right) × 123.75 mm (foot to head), in-plane resolution 3.75 mm × 3.75 mm, 25 axial slices, slice-thickness 3.75 mm, interslice-gap 1.25 mm, 112 repetitions per run after 10 dummy acquisitions. Total duration per run was approximately 5 min.

### fMRI preprocessing

See Supplementary Information for the description of fMRI preprocessing.

### Dynamic causal modeling

Dynamic causal modeling (DCM) [43] was used to measure the ECs elicited by the cocaine-word Stroop fMRI task [18, 19] following acute oral administration of placebo or mirtazapine with experimenters blinded to the drug condition until the initiation of data analysis. fMRI-based DCM is a biophysical model of the underlying neuronal connectivity and how the neuronal connectivity generates the observed BOLD signal [43]. DCM with the deterministic option, as implemented in Statistical Parametric Mapping 12 (SPM12) software (Revision 7219; http://www.fil.ion.ucl.ac.uk/spm/) was used for EC analysis. The use of DCM in drug-related, attentional bias studies has been described [18, 19].

### Candidate a priori DCM nodes

Based on studies suggesting similarity in attentional bias for drug cues across drug classes [18, 44–46], we used left (L) and right (R) ACC, L and R medial orbitofrontal cortex (MOFC), L and R posterior cingulate cortex (PCC), L and R insula, L and R hippocampus, and L and R striatum as the a priori selected DCM nodes as recently published [19]. The DCM nodes were specifically constrained by the task-related brain activation (see Supplementary Information) in two steps: (1) to determine if a candidate DCM node was selected as a final DCM node (to be selected, the brain region corresponding to the candidate DCM node needed to exhibit at least 10 active voxels on the brain activation found by any of the SPM second level analyses), and (2) to localize the position of each selected DCM node, i.e., to use the voxel with the local maximum t value as the center of the sphere (the DCM node). After constraint by the brain activations found in this study, the following six brain regions were selected as final DCM nodes: (1) L-ACC (*x* = −4, *y* = 47, *z* = 4); (2) R-MOFC (*x* = 4, *y* = 51, *z* = −11); (3) L-PCC (*x* = −8, *y* = −56, *z* = 20); (4) L-insula (*x* = −34, *y* = −19, *z* = 5); (5) R-hippocampus (*x* = 31, *y* = −13, *z* = −20); and (6) R-putamen (*x* = 20, *y* = 6, *z* = −6). Each node was a sphere with 6 mm radius, and the *x*, *y*, *z* values (in mm) are the MNI coordinates of the center of each node determined by the *t*-test maximum within the fMRI activation cluster corresponding to that node. For each node, the functional activation BOLD time-series, which was used for DCM analysis, was extracted using the previous methods [47, 48].

### Driving/modulatory inputs for the DCM analyses from placebo scans

Based on two types of task blocks (CW and NW), two parametric regressors, called “Placebo All-Words” and “Placebo CW-minus-NW,” respectively, were created for the DCM analyses. The All-Words regressor was All Words (CW and NW) minus implicit baseline, and the CW-minus-NW regressor was CW minus NW. In other words, the first regressor was non-specific word effects, relative to the implicit baseline, while the second modeled the special effect of CW over NW. The All-Words regressor was used as a single input to all the nodes of the DCM (driving input), and the CW-minus-NW regressor was used as a putative modulator (modulatory input) of all ECs (i.e., a modulatory input is an experimental factor eliciting change in EC). The detailed method of constructing these regressors can be found online (https://github.com/HaukeHillebrandt/SPM_connectome).

### Driving/modulatory inputs for the DCM analyses testing the effects of mirtazapine

In a separate DCM analysis, the effects of mirtazapine were evaluated based on the contrast between the mirtazapine CW-modulation (similarly defined as above for the placebo scan) and the placebo CW-modulation. Towards that end, the placebo and mirtazapine scans were combined using the SPM12 command “spm_fmri_concatenate”. Then, two DCM parametric regressors, called “Two-scan All-Words” and “mirtazapine-minus-placebo,” respectively, were created for these DCM analyses. The “Two-scan All-Words” parametric regressor reflects the common features of the CW and NW in both placebo and mirtazapine scans, and was used as a single input to the DCM (driving input). The mirtazapine-minus-placebo parametric regressor was defined as the mirtazapine CW-modulation minus the placebo CW-modulation. This parametric regressor reflects the specific effects of mirtazapine CW-modulation over placebo CW-modulation. In this study, the change of EC (relative to the endogenous connectivity) for the mirtazapine-CW modulation minus placebo CW-modulation is termed as “mirtazapine-minus-placebo modulatory change”.

### Parametric Empirical Bayes (PEB) analysis to detect most parsimonious EC model

**Fig. 1:**
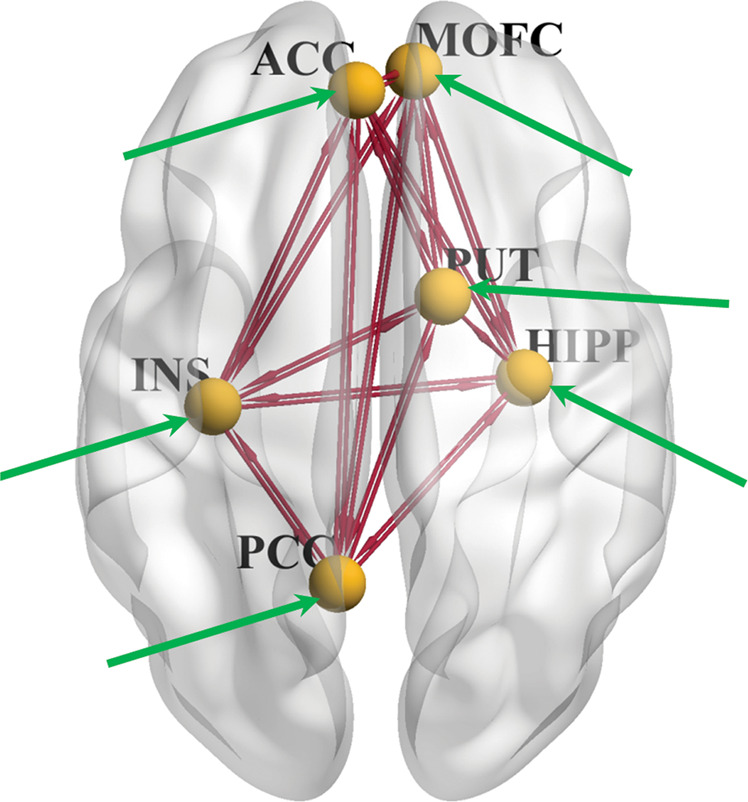
The initially fully connected model, visualized with the BrainNet Viewer (http://www.nitrc.org/projects/bnv/) [71]. The six DCM nodes are illustrated as gold spheres, and the dark red lines with arrows represent the endogenous connectivities. The All-Words driving input (green line with arrow) affected all six nodes, and the modulator (e.g., mirtazapine CW-modulator) affected all endogenous connectivities. Whether the driving input effect to a node (or the modulatory effect on an EC) was different from zero was determined based on the posterior probability (see Results). ACC, anterior cingulate cortex; MOFC, medial orbitofrontal cortex; INS, insula; HIPP, hippocampus; PCC, posterior cingulate cortex; PUT, putamen. The left side of this figure aligns with the left brain hemisphere.

After specifying an initial fully connected model (i.e., there were two bidirectional endogenous connectivities between any two nodes, the driving input affected all the DCM nodes, and the modulatory input affected all the endogenous connectivities, see Fig. 1), the PEB analysis [49], as implemented in DCM for SPM12 (Revision 7219), was used to conduct DCM group level analyses for the EC parameters. Corresponding to the three hypotheses (i.e., the replication of the positive association between ACC → hippocampus EC and attentional bias; the magnitude of the cue-related ACC → hippocampus EC would be reduced after mirtazapine administration; this mirtazapine-induced ACC → hippocampus EC reduction would be greater in the CUD participants with wild-type *HTR2C* gene compared to those with *HTR2C* SNP, the following PEB analyses were conducted: (1A) test the placebo CW-modulation (vs. zero) across all participants; (1B) test the linear regression of placebo CW-modulation on attentional bias across all participants; (2) test the mirtazapine-minus-placebo modulatory change (vs. zero) across all participants; and (3) test the difference in mirtazapine-minus-placebo modulatory change between the participants with the wild-type *HTR2C*and the *HTR2C* SNP. Here, an EC finding was considered reliable (e.g., a modulatory effect is different from zero) if Bayesian posterior probability (PP) > 0.95 (corresponding to a Bayes-factor of 3). See Supplementary Information for more information about PP and the advantages of DCM-PEB posterior inferences, including the avoidance of multiple-comparison problem [43, 50, 51]. In DCM, EC is in unit of hertz (Hz) because it is rate of change (rate constants) [52]. For example, placebo CW-modulation is the rate of change in the EC due to the modulatory inputs (CW relative to NW).

## Results

### Non-imaging results

As illustrated in Table 1, there were no significant differences in demographic or substance use parameters, difference in abstinence time between the first and second scans (three participants had missing data), route of cocaine administration, nor the number of days between the placebo and mirtazapine scans for the wild-type and *HTR2C*SNP subgroups. The frequency (~50%) of the *HTR2C* SNP in our largely African American pool was higher than expected in the general population (~20%) (1000 Genome [53]). Among all the 28 participants, 22 smoked cocaine and six used cocaine intranasally (Table 1). All participants exhibited clean urines prior at both visits. Table 2 shows that baseline values in accuracy in the task based, cocaine-word Stroop task were comparable across the entire sample, indicating that there were no obvious evidence of withdrawal symptoms (e.g., hypersomnia) in the participants who were negative for cocaine and other substances [54, 55]. Attentional bias did not differ in participants with the wild-type and *HTR2C* SNP during the placebo (*t*_26_ = 0.55, *p* = 0.59) or mirtazapine scan (*t*_26_ = 1.29, *p* = 0.21), nor was the change of attentional bias from placebo scan to mirtazapine scan different (*t* = 0.67, df = 26, *p* = 0.51). Non-parametric statistical methods were used to analyze accuracy which is non-Gaussian distributed (see Supplementary Information for details).

**Table 2:** In-scanner behavior under placebo and mirtazapine conditions.

	All (*n* = 28)	Wild-type *HTR2C* (*n* = 15)	*HTR2C* SNP (*n* = 13)
In-scanner Cocaine-Word Stroop Performance after Placebo			
Attentional bias (ms; mean±S.D.)	^a^2.72 ± 41.1 *t*_27_ = 0.35, *p* = 0.73	^b^−1.32 ± 37.4 *t*_14_ = 0.14, *p* = 0.89	^c^7.38 ± 46.0 *t*_12_ = 0.58, *p* = 0.57
Accuracy (%) during CW and NW trials in median (IQR)	98.33% (3.75%)	97.50% (5.83%)	98.33% (3.33%)
Accuracy (%) during CW trials in median (IQR)	98.33% (3.33%)	98.33% (5.00%)	98.33% (3.33%)
Accuracy (%) during NW trials in median (IQR)	96.67% (7.50%)	96.67% (8.33%)	96.67% (8.33%)
In-scanner Cocaine-Word Stroop Performance after Mirtazapine^***a***^			
Attentional bias (ms; mean±S.D.)	^a^11.89 ± 58.2 *t*_27_ = 1.08, *p* = 0.29	^b^−1.14 ± 59.5 *t*_14_ = 0.07, *p* = 0.94	^c^26.91 ± 55.1 *t*_12_ = 1.76, *p* = 0.10
Accuracy (%) during CW and NW trials in median (IQR)	93.33% (8.75%)	95.00% (6.25%)	93.33% (8.33%)
Accuracy (%) during CW trials in median (IQR)	95.00% (8.33%)	95.00% (8.33%)	95.00% (12.5%)
Accuracy (%) during NW trials in median (IQR)	93.33% (8.33%)	96.67% (6.67%)	93.33% (9.17%)
Change (∆) of attentional bias from Placebo to Mirtazapine			
∆ Attentional bias (ms; mean ± S.D.)	^a^9.16 ± 62.31 *t*_27_ = 0.78, *p* = 0.44	^b^0.18 ± 70.04 *t*_14_ = 0.01, *p* = 0.99	^c^19.52 ± 52.87 *t*_12_ = 1.10, *p* = 0.28

Please see text for additional statistical outcomes of the corresponding two-sample Student’s *t* test analyses testing the differences between the group expressing the wild-type HTR2C and the group expressing the HTR2C SNP.

^a^One-sample Student’s *t* test was used to test whether attentional bias was significantly different from zero across all participants.

^b^One-sample Student’s *t* test was used to test whether attentional bias was significantly different from zero across the participants expressing the wild-type *HTR2C.*

^c^One-sample Student’s *t* test was used to test whether attentional bias was significantly different from zero across the participants expressing the *HTR2C* SNP.

### Brain activation results for localizing DCM nodes

**Fig. 2:**
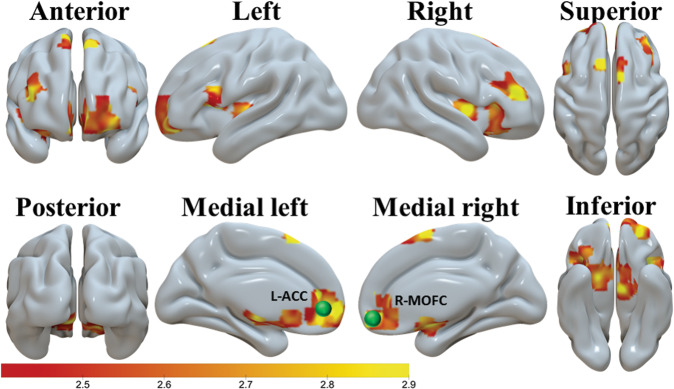
The brain activation used to constrain the a priori-selected L-ACC (in medial left view) and R-MOFC (in medial right view) DCM node is depicted using the Surf Ice software (https://www.nitrc.org/plugins/mwiki/index.php/surfice:MainPage, posted by Dr. Chris Rorden). The brain activation clusters shown were identified by a SPM second level one-sample *t*-test analysis for the placebo scan and for all participants (CW minus NW > 0), with cluster-defining threshold *t* = 2.4 and uncorrected two-tailed cluster level *p* < 0.05. Scale on the color bar represents voxel t values. The nodes (spheres) are larger than the exact ones for demonstration purpose.

**Fig. 3:**
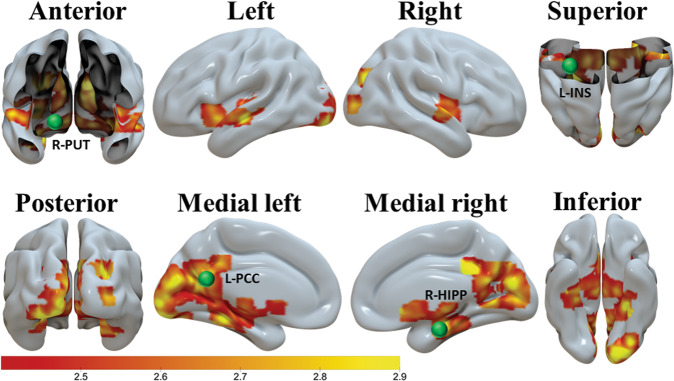
The brain activation used to constrain the a priori-selected R-putamen (in anterior cut view), L-INS (in superior cut view), L-PCC (in medial left view), and R-hippocampus (in medial right view) DCM nodes. These brain activations were found by a SPM second level *t*-test comparison of the two genotype groups, with cluster-defining threshold *t* = 2.4 and uncorrected two-tailed cluster level *p* < 0.05. This analysis found that, during the mirtazapine scan, the participants with the wild-type *HTR2C*exhibited greater cocaine word-elicited activation than those with the *HTR2C* SNP. Scale on the color bar represents voxel *t* values. The nodes (spheres) are larger than the exact ones for demonstration purpose.

The brain activation results used to constrain the DCM nodes are shown in the Supplementary Information. See Fig. 2 and Supplementary Table 2 for detailed information regarding the brain activations used to constrain the L-ACC and the R-MOFC nodes. See Fig. 3 and Supplementary Table 3 for detailed information regarding additional brain activations used to constrain the remaining four DCM nodes.

### DCM-PEB connectivity results

The following PEB analyses were conducted in correspondence with our three hypotheses: (1) the positive association between ACC → hippocampus EC and attentional bias will be replicated; (2) the magnitude of the cue-related ACC → hippocampus EC will be reduced after mirtazapine administration; (3) mirtazapine-induced reduction in ACC → hippocampus EC will be greater in the CUD participants with wild-type *HTR2C* gene compared to those with *HTR2C* SNP. As mentioned above, the PEB analysis employs Bayesian posterior inference and eschews the multiple-comparison problem because of the lack of false positives without the need to correct the EC results for multiple comparisons.

### PEB analysis for hypothesis #1

**Fig. 4:**
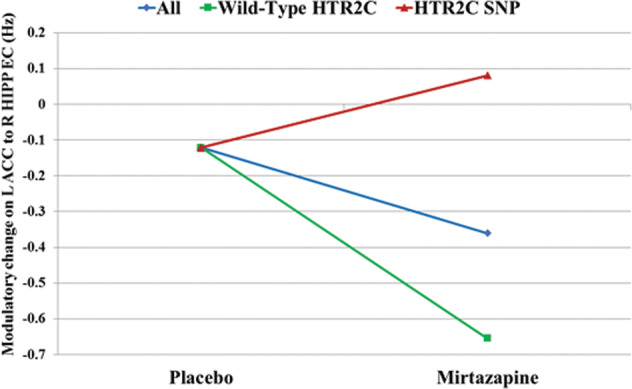
The mean CW modulatory change of the L-ACC → R-hippocampus (HIPP) EC is illustrated for the *HTR2C* SNP subgroup (red line), wild-type *HTR2C* subgroup (green line), and all participants (combined *HTR*2C SNP and wild-type HTR2C subgroups) (blue line) upon placebo and mirtazapine scans. The differences between the mirtazapine and placebo scans (mirtazapine minus placebo) were all reliable (PP = 1) with 0.2028 Hz, −0.5327 Hz, and −0.2390 Hz for the *HTR2C* SNP, wild-type *HTR2C*, and all participants, respectively [54, 55, 72–74].

We first tested if the mean of placebo CW-modulation was different from zero (Hypothesis #1A) for each EC across all participants. For each EC, the mean placebo CW-modulation and the corresponding PP are shown in Supplementary Table 4. As shown in Fig. 4, and Supplementary Table 4, the mean placebo CW-modulation of the L-ACC → R-hippocampus EC (i.e., the hypothesized EC) was −0.1218 Hz, with PP = 1.We then tested the linear regression of the placebo CW-modulation on attentional bias (ΔRT) for each EC and across all the participants (Hypothesis #1B). For each EC, the regression slope (or regression coefficient) *beta* and the corresponding PP are shown in Supplementary Table 4. The regression of the placebo CW-modulation of the L-ACC → R-hippocampus EC (i.e., the hypothesized EC) on attentional bias scores exhibited a reliable positive regression slope (regression parameter *beta* = 0.0029, PP = 1. The positive linear relationship between the placebo CW-modulation of the L-ACC → R-hippocampus EC and attentional bias suggests that reduction of the CW-modulation of the L-ACC → R-hippocampus EC has the potential to reduce attentional bias.

### PEB analysis for test of hypothesis #2

We tested if the mirtazapine-minus-placebo modulatory change was different from zero for each EC across all participants. For each EC, the mean mirtazapine-minus-placebo modulatory change and the corresponding PP are shown in Supplementary Table 4. Specifically, the mean mirtazapine-minus-placebo modulatory change in the L-ACC → R-hippocampus EC (i.e., the hypothesized EC) was negative with strong confidence (−0.2390 Hz; PP = 1), suggesting a reliable mirtazapine-related decrease of CW-modulation of the L-ACC → R-hippocampus EC for all participants (Fig. 4, blue line). Because the mirtazapine-minus-placebo modulatory change was relative to the placebo CW-modulation (baseline − 0.1218 Hz), the CW modulatory change upon mirtazapine scan was therefore −0.3608 Hz as shown in Fig. 4 (blue line). See Supplementary Information for the non-hypothesized ECs which were altered by the mirtazapine and also showed reliable (PP = 1) linear relationships between placebo CW-modulation and attentional bias.

### PEB analysis for hypothesis #3

We tested if the difference of the placebo CW-modulation for each EC differed between the participants with the wild-type and *HTR2C* SNP. For each EC, the group difference on mean placebo CW-modulation and the corresponding PP are shown in Supplementary Table 5. Specifically, the mean of the placebo CW-modulation of the L-ACC → R-hippocampus EC was identical (−0.1218 Hz) for both wild-type and *HTR2C* SNP participants (Fig. 4). The difference of the mirtazapine-minus-placebo modulatory change for each EC was also assessed between the participants carrying the wild-type and *HTR2C* SNP. For each EC, the group difference (*HTR2C* SNP minus wild-type *HTR2C*) of mean placebo CW-modulation and the corresponding PP are shown in Supplementary Table 5. In particular, the group difference (*HTR2C* SNP minus wild-type *HTR2C*) of the mirtazapine-minus-placebo modulatory change of the L-ACC → R-hippocampus EC was 0.7355 Hz with PP = 1. In two *post hoc* analyses, we measured the mirtazapine-minus-placebo modulatory change separately in each of the two groups (*HTR2C* SNP and wild-type *HTR2C*). These *post hoc* analyses indicated that the mirtazapine CW-modulation was 0.0810 Hz for the*HTR2C* SNP group and −0.6545 Hz for the wild-type *HTR2C* group as shown in Fig. 3, suggesting that the decreased mirtazapine-minus-placebo modulatory change of L-ACC → R-hippocampus EC (−0.2390 Hz, PP = 1; found in the PEB analysis for test of hypothesis #2) was mainly seen in the wild-type *HTR2C* group (−0.6545 Hz for the wild-type *HTR2Cvs*. 0.0810 Hz for*HTR2C* SNP group).

## Discussion

A primary challenge is to integrate brain, behavior, and genetic substrates of CUD to ultimately craft strategies to rectify the propensity to relapse during recovery. The present study objectively evaluated attentional bias toward cocaine-associated cues within the context of functional connectivity and a pharmacogenetic analysis in CUD participants. Using Bayesian posterior inference, we found that the L-ACC → R-hippocampus EC under the placebo condition positively associated with attentional bias, replicating previous findings [18, 19], and uncovered unique ECs of interest (see additional Results/Discussion in Supplementary Information). Intriguingly, compared to placebo, mirtazapine decreased the L-ACC → R-hippocampus EC in tandem with reduced attentional bias for all CUD participants, with effectiveness identified as greater in CUD participants carrying the wild-type *HTR2C*. These results are distinctive in outlining a deeper description of the complex neurocircuitry, behavior and pharmacogenetic profile of CUD participants [3, 9, 56].

We found that, across all CUD participants in the present study, the L-ACC → R-hippocampus EC positively associated with attentional bias, replicating outcomes in a different CUD participant cohort [18] and in an opioid use disorder sample [19]. We interpret this replicated, positive linear relationship as reflecting individual differences in one or more brain processes including, but not limited to, retrieval of hippocampus-mediated episodic memories related to drug use [18, 19], the level of drug cue-related plasticity in the hippocampus [19, 57], and/or the transformation of reward-related information from ACC → hippocampus [58]. The findings in the current study are consistent with our hypothesis that the ACC → hippocampus EC may be a neurocircuit related to drug cue reactivity generalized across substance use disorders [19]. We speculate that this neurocircuit may serve as a key brain substrate of craving in response to drug-associated cues.

The 5-HT_2A/2C_R antagonist mirtazapine decreased the L-ACC → R-hippocampus EC in tandem with reduced attentional bias for all CUD participants, with outcomes predicated on the *HTR2C* genotype. Given that cellular studies indicate reduced functionality of the 5-HT_2C_R carrying the rs6318 SNP [38, 39], the present observations suggest that mirtazapine effectiveness to suppress attentional bias-associated L-ACC → R-hippocampus EC is linked to the degree of 5-HT_2C_R functionality. Relatedly, CUD participants with the rs6318 SNP exhibit significantly higher attentional bias toward drug cues relative to carriers of the wild-type gene [40], and may be more resistant to mirtazapine in the absence of a maximally functional 5-HT_2C_R. Although speculative, this line of thought is consistent with observations that describe the interactivity of the 5-HT_2A_R and 5-HT_2C_R systems in vivo and in vitro (see Introduction) [16, 17]. Furthermore, we demonstrated that a selective 5-HT_2C_R antagonist augmented cocaine-mediated behaviors in rats, an outcome which is lost with repeated administration of cocaine [59]. These data suggest that 5-HT_2C_R systems may be dysregulated in carriers of the rs6318 SNP coupled to conditions of repeated cocaine administration which is likely the case for the CUD participants who have ~14 years of lifetime cocaine use. These data may be interpreted to suggest that the wild-type 5-HT_2C_R is necessary for the efficacy of mirtazapine to decrease activated EC in CUD participants. While future studies are required to further interrogate the interrelationship between 5-HT_2C_R and 5-HT_2A_R systems in drug-related behaviors in humans with CUD, our present results suggest that mirtazapine may serve as an abstinence enhancer to mitigate brain substrates of craving in response to cocaine-associated stimuli particularly for participants with the normal function *HTR2C*.

Reliable effects of mirtazapine were observed at the EC level; however, the effects of mirtazapine were not observed at the behavioral level, and at the contrast-elicited univariate brain activation level. The lack of statistically significant univariate brain activation results at the group level is consistent with previous studies investigating drug-cue related attentional bias [18, 60]. The reliable effects of mirtazapine on EC from the DCM analysis are consistent with the theory that the brain connectivity approach is more sensitive than univariate activation analysis in detecting neuronal alterations in neurological disorders [61]. The findings on the ACC → hippocampus EC are consistent with the results found in a separate CUD population [18] and in a population with opioid use disorder [19]. Considering findings that individual differences in attentional bias have been predictive of treatment dropout and relapse within CUD treatment populations [45, 62], we believe that our EC findings may have important clinical implications and support further research on the utility of DCM in detecting effects of the treatment.

These findings should be interpreted within several limitations. (1) Both within-group [18, 60, 63, 64] and between-group designs [65–68] have been used to study drug cue reactivity. The present study employed a within-group design as non-drug users are unlikely to exhibit attentional bias toward cocaine-associated cues. However, larger studies employing strategies such as drug images and/or tactile stimuli, or actual gaze-fixation as captured by eye-tracking [69] provide assays with perhaps greater saliency than drug-related-words in the study of attentional bias in the future. (2) The brain activation and the EC modulatory changes were based on the contrast between CW trials and NW trials in a block design, and thus may have been slightly confounded by the sporadic incorrect responses (less than 9% for all trials) [62]. (3) As discussed previously [18], a limited number of nodes are practical in DCM analysis, but inclusion of additional DCM nodes will allow for more complete interrogation of neurocircuitry important for cocaine cue reactivity in the future. (4) Mirtazapine is a potent 5-HT_2A_R/5-HT_2C_R antagonist, but does exhibit affinity for histamine and norepinephrine receptors, actions that could contribute to the observed outcomes through central or peripheral actions, including sedative effects [70]. (5) The sample size was small for the between-group analysis. However, the findings related to the ACC → hippocampus EC are consistent with the results found across samples investigating CUD [18] and opioid use disorder [19]. This consistency reduces the likelihood that the results of the between group analysis are chance findings, and future studies will expand on our initial findings.

In summary, the current study replicated the previous findings that the ACC → hippocampus EC is positively associated with drug cue related attentional bias. Importantly, mirtazapine decreased this ACC → hippocampus EC, with greater effectiveness in those CUD participants carrying the wild-type 5-HT_2C_R gene relative to the 5-HT_2C_R SNP rs6318. These results implicate serotonergic substrates that underlie sensitivity to cocaine-associated stimuli in CUD participants with a specific pharmacogenetic descriptor, outlining a richer “fingerprint” of the complex neurocircuitry, behavior and pharmacogenetics profile of CUD participants which may provide important implication for future medications development for CUD.

## Supplementary information

Supplemental Material
