# Supplementary material for: A serotonergic biobehavioral signature differentiates cocaine use disorder participants administered mirtazapine
Source: Transl Psychiatry. 2022 May 6;12:187. doi: 10.1038/s41398-022-01934-w (PMC9076859; doi:10.1038/s41398-022-01934-w)
Supplement: Supplementary file 1 — Supplemental Material [file 41398_2022_1934_MOESM1_ESM.docx]

Supplementary Information

A Serotonergic Biobehavioral Signature Differentiates Cocaine Use Disorder Participants Administered Mirtazapine

Liangsuo Ma^1,2#*^, Kathryn A. Cunningham^5#*^, Noelle C. Anastasio^5^, James M. Bjork^1,2^, Brian A. Taylor^1,3^, Albert J. Arias^1,2^, Brien P. Riley^4^, Andrew D. Snyder^1^, and F. Gerard Moeller^1,2^

^1^Institute for Drug and Alcohol Studies, ^2^Department of Psychiatry, ^3^Department of Biomedical Engineering,^4^Virginia Institute for Psychiatric and Behavioral Genetics

Virginia Commonwealth University, Richmond, Virginia, USA

^5^Center for Addiction Research and Department of Pharmacology and Toxicology

University of Texas Medical Branch, Galveston, Texas, USA

^#^ CO-FIRST AUTHORS

*** Correspondence:**

Liangsuo Ma, Ph.D.

Institute for Drug and Alcohol Studies,

Department of Psychiatry

Virginia Commonwealth University

203 East Cary Street, Suite202, Richmond, Virginia 23219, USA

Phone: +1 804-828-2871; Fax: +1 804-827-2565

Email: [Liangsuo.ma@vcuhealth.org](mailto:Liangsuo.ma@vcuhealth.org)

*** Correspondence:**

Kathryn A. Cunningham, Ph.D.

Center for Addiction Research

University of Texas Medical Branch

301 University Boulevard, Galveston, Texas 77555, USA

Phone: +1-409-772-9642; FAX 409-747-7050

Email: [kcunning@utmb.edu](mailto:kcunning@utmb.edu)

## ADDITIONAL METHODS

## Cocaine-word Stroop task. We employed the cocaine-word Stroop task within the fMRI scanner to assess attentional bias towards cocaine-related stimuli as we have previously described ^1^. Four 30-second (30-s) cocaine word (CW; e.g., cocaine, crack) and four 30-s neutral word (NW; kitchen, table) blocks alternated with a 6-s pause between blocks with each word randomly printed in one of three different colors (blue, green, red). The participant was instructed to ignore the meaning of the word and to press the appropriately colored button on the MRI-compatible response box to denote the color in which the word was printed. For each participant, the mean reaction times (RTs) during the CW blocks and during the NW blocks were computed.

## fMRI Preprocessing. fMRI voxels in which the signal in a particular volume exceeded plus or minus four standard deviations from the mean of the run were considered to be outliers and data were imputed as the mean of the two nearest non-outlier neighbors using the Analysis of Functional NeuroImages (AFNI) ^2^ module “3dDespike” (http://afni.nimh.nih.gov/afni/). All subsequent preprocessing used SPM12 software (implemented in Matlab R2015b, Mathworks Inc. Sherborn MA, USA). After slice timing corrections, the fMRI series was realigned to correct for head motion. Runs with head motion greater than one voxel (3.75 mm translation on any axis) or rotation greater than 3.75 degrees were removed from the analysis. The anatomical image was coregistered to the fMRI images and spatially transformed to the Montreal Neurological Institute (MNI) standard atlas coordinates using the SPM12 Normalise module with the SPM12 tissue probability maps. The transformation parameters were applied to the fMRI images, which were then resliced to 2 mm isotropic resolution and spatially smoothed with a Gaussian filter of 8 mm isotropic full width at half maximum.

## Task-related brain activation used to constrain the DCM nodes. Selected DCM nodes were based upon task-related brain activation. The spatial extent of the candidate DCM nodes was constrained by task contrast activation in the second-level SPM12 analysis of the group under placebo conditions. The first-level univariate statistical analysis of the fMRI data was conducted using SPM12 for both placebo and mirtazapine scans. CW and NW blocks (conditions) were modeled by boxcar functions convolved with the SPM12 canonical hemodynamic response function. The parameters for each condition were estimated using the General Linear Model ^3^ at each voxel without global normalization. The fMRI time series was high-pass filtered with an optimized cut-off period of 144 s determined by the Fourier transformation of each condition’s time model. At each voxel, activation was measured as the contrast of the parameter estimate for CW blocks minus the parameter estimate for NW blocks (CW minus NW contrast). The resulting set of voxel values for this contrast constitutes a statistical parametric map (contrast image). The CW minus NW contrast image (one per participant) was then entered at the SPM12 second level (i.e., random effects) group analysis.

Each of the two pharmacological conditions was separately analyzed with a one-sample *t*-test to determine the group level BOLD activation using an SPM12 second level Random Effects^4^. In addition, a two-sample *t*-test comparison of the two genotype groups was conducted for each pharmacological condition. Furthermore, a second level paired *t*-test was conducted to test the difference in BOLD activation between the mirtazapine and placebo conditions. The univariate brain activations were used to constrain the spatial extent of the *a priori*-selected DCM nodes in this study to the most task-relevant (and least noisy) voxels. Therefore, a less conservative cluster-defining threshold (t = 2.4) was used, and the regional brain activation was defined as the uncorrected cluster probability (*p*) less than 0.05 (two tail). It is commonly accepted that less conservative statistical criteria can be used when brain activations are used for determining the extent of DCM nodes.^5^Anatomical labels for regions of activation were determined using the Anatomical Automatic Labeling (AAL2) toolbox ^6^.

**Concatenation of placebo and mirtazapine runs in the DCM analyses.** It is common to concatenate and extract timeseries from multiple fMRI runs for connectivity analyses such as DCM. This strategy is employed because the SPM time series which are used for DCM analysis are extracted on a per-session basis. In this study, the modulation effects of the cocaine words (CW) were measured relative to the neutral words (NW) (i.e., CW-minus-NW modulator). Since the neuropharmacological effects of mirtazapine (or placebo) on the CW and NW signals should be similar, any effects of mirtazapine and placebo should have been cancelled out by the difference between CW and NW in the CW-minus-NW modulator. Alternatively, we can compare the EC between the mirtazapine and placebo conditions using a factorial design. Compared to the concatenated approach in which we only estimated the difference in the CW-minus-NW modulator between mirtazapine and placebo, the number of DCM parameters to be estimated in the approach using a factorial design is double in the PEB framework (average of the CW-minus-NW modulator across mirtazapine and placebo AND difference in the CW-minus-NW modulator between mirtazapine and placebo). Given the small sample size, we opted to employ the concatenated approach.

**The advantages of DCM-PEB posterior inferences.** The advantage of the regression analysis within the DCM-PEB framework is that the covariance among DCM parameters is automatically taken into consideration. DCM-PEB conducts group level analyses using Bayesian posterior inference ^7^. In these posterior inferences, posterior probability (PP) is used as an indicator of the confidence or reliability in whether a modulatory change in a group is different from zero (or different from modulatory change in another group) or the confidence in the degree of linear relationship between variables. A key advantage of employing Bayesian posterior inference is the lack of false positives, thus removing the need to correct for the multiple comparison problem ^5^. The PP (0≤PP≤ 1) is the conditional probability that is computed by DCM-PEB after the available information (such as prior probability and likelihood function) is considered by the Bayesian analysis. The higher the PP, the greater the confidence. Here, an EC finding was considered reliable if Bayesian-PP>0.95 (corresponding to a Bayes-factor of 3). Bayesian posterior inference during the PEB analyses eschews the multiple comparison problem because of the lack of false positives ^5, 7-8^. Specifically, the PP of an EC, given the data, is the same, regardless of whether all ECs in the same DCM were analyzed or not ^7^. This is implemented by using independent prior distributions for different ECs ^7^.

## ADDITIONAL RESULTS

**In-scanner percentage of correct responses (i.e., accuracy)during task.** Normality Tests ^9^ indicated that the accuracy was non-normal distributed (ten tests, the biggest p = 0.01). Analyses on the repeated measures (accuracy during CW trials vs. accuracy during NW trials) using non-parametric Friedman tests indicated that the accuracy was not significantly different between the CW and NW trials during the placebo scan for the entire sample (Chi-square [1,55]=1.32, *p*=.2513), the participants with the wild-type *HTR2C* (Chi-square [1,29]=1, *p*=.3173) and participants with the *HTR2C* SNP (Chi-square [1,25]=0.4, *p*=.5271). Similarly, the accuracy was not significantly different between the CW and NW trials during the mirtazapine scan for the entire sample (Chi-square [1,55]=1.38, *p*=.2393), participants with the wild-type *HTR2C* (Chi-square [1,29]=1.67, *p*=.1967) and the *HTR2C* SNP (Chi-square [1,25]=0.09, *p*=.7630). Therefore, accuracy shown in the subsequent analyses was measured across both CW and NW trials.

Non-parametric Friedman tests on the repeated measures (mirtazapine vs. placebo) indicated that the accuracy was significantly lower during the mirtazapine scan than the placebo scan for the entire sample (Chi-square [1,111]=11.26, *p*=.0008), and participants with the wild-type *HTR2C* (Chi-square [1,59]=11.56, *p*=.0007). But, the accuracy was similar during the mirtazapine and placebo scans for the participants with the *HTR2C* SNP (Chi-square [1,51]=1.64, *p*=.2008). Kruskal-Wallis tests showed that the participants with the wild-type *HTR2C* and the *HTR2C* SNP did not differ in accuracy during the placebo scan (Chi-square [1,55]= 0.12, *p*=.7309), the mirtazapine scan (Chi-square [1,55]= 0.93, *p*=.3338), or both placebo and mirtazapine scans combined (Chi-square [1,111]= 0.53, *p*=.4648).

**Contrast-elicited brain activation results for localizing DCM nodes.** None of the SPM univariate activation analyses described in the Methods identified any statistically significant clusters of activation when a conservative cluster forming threshold (voxel *p*=0.001) was used and when family wise error (FWE) or false discovery rate (FDR) two-tailed cluster level *p*<0.10 was used. However, it is commonly accepted that less conservative statistical criteria can be used when brain activations are used for constraining the extent of DCM nodes ^5, 10^.

When a less conservative statistical criteria (cluster-defining threshold t=2.4 which corresponds to p=0.01 and uncorrected two-tailed cluster level *p*<0.05) was used, the spatial extents of the *a priori*-selected candidate DCM nodes were best constrained (in terms of voxel t-value and number of voxels) by the brain activations from two SPM second level univariate analyses. Specifically, the L-ACC (left anterior cingulate cortex) and the R-MOFC (medial orbitofrontal cortex) nodes were constrained by the activation clusters (CW minus NW>0) found by the second level univariate SPM one-sample *t*-test analysis for the placebo scan and for all the participants. See Figure 1 and Supplementary Information [SI]-Table 2 for detailed information regarding these clusters. The remaining four DCM nodes were constrained by a two-sample *t*-test comparison of the two genotype groups showing that, during the mirtazapine scan, the participants with the wild-type *HTR2C*exhibited greater cocaine word-elicited activation than those with the *HTR2C* SNP in several clusters. See Figure 2 and SI-Table 3 for detailed information regarding these clusters. To reiterate, the activation clusters described above were only used to constrain the extent of the *a priori*-selected candidate DCM nodes.

**The DCM results related to the non-hypothesized ECs showing reliable positive linear relationships between placebo CW-modulation and attentional bias.** Four non-hypothesized ECs showed reliable (PP=1) positive linear relationships between placebo CW-modulation and attentional bias (SI-Figure 1, left panel), and these ECs also showed reliable (PP=1) mirtazapine-minus-placebo modulatory changes. Specifically, the ECs between R-MOFC and L-INS and the EC from R-hippocamus to R-MOFC showed a reliable reduced mirtazapine-minus-placebo modulatory change (SI-Figures 1, middle panel). The non-hypothesized L-insula to R-hippocampus EC showed reliable increased mirtazapine-minus-placebo modulatory change (SI-Figures 1, right panel).

**The DCM results related to the non-hypothesized ECs showing reliable negative linear relationships between placebo CW-modulation and attentional bias.** Four non-hypothesized ECs showed reliable (PP=1) negative linear relationships between placebo CW-modulation and attentional bias (SI-Figure 2, left panel), and these ECs also showed reliable (PP=1) mirtazapine-minus-placebo modulatory changes. Specifically, the ECs from L-ACC to L-INS, from L-PCC to R-MOFC, and from R-putaman to R-MOFC showed a reliable increased mirtazapine-minus-placebo modulatory change (SI-Figures 2, middle panel). The non-hypothesized L-insula to L-ACC EC showed reliable reduced mirtazapine-minus-placebo modulatory change (SI-Figures 2, right panel).

## ADDITIONAL DISCUSSION

**Discussion of non-hypothesized ECs.** Eight non-hypothesized ECs showed reliable mirtazapine-minus-placebo modulatory change reflecting the effects of mirtazapine on EC relative to the placebo, and a reliable linear relationship was observed between placebo CW-modulation and attentional bias. The impact of mirtazapine pretreatment on most of these ECs (seven out of nine, including the hypothesized ECs) were related to reduced attentional bias. Interestingly, five out of these nine ECs target the MOFC, and the effects of mirtazapine on these five ECs were related to reduced attentional bias. This finding is consistent with the role of OFC in drive and compulsive repetitive behaviors ^11^. As shown in SI-Table 4, there are ECs described such as the R-hippocampus to L-ACC EC affected by mirtazapine, but unrelated to attentional bias and ECs (e.g., R-putamen to L-ACC EC) related to the attentional bias, but not affected by the mirtazapine.

**
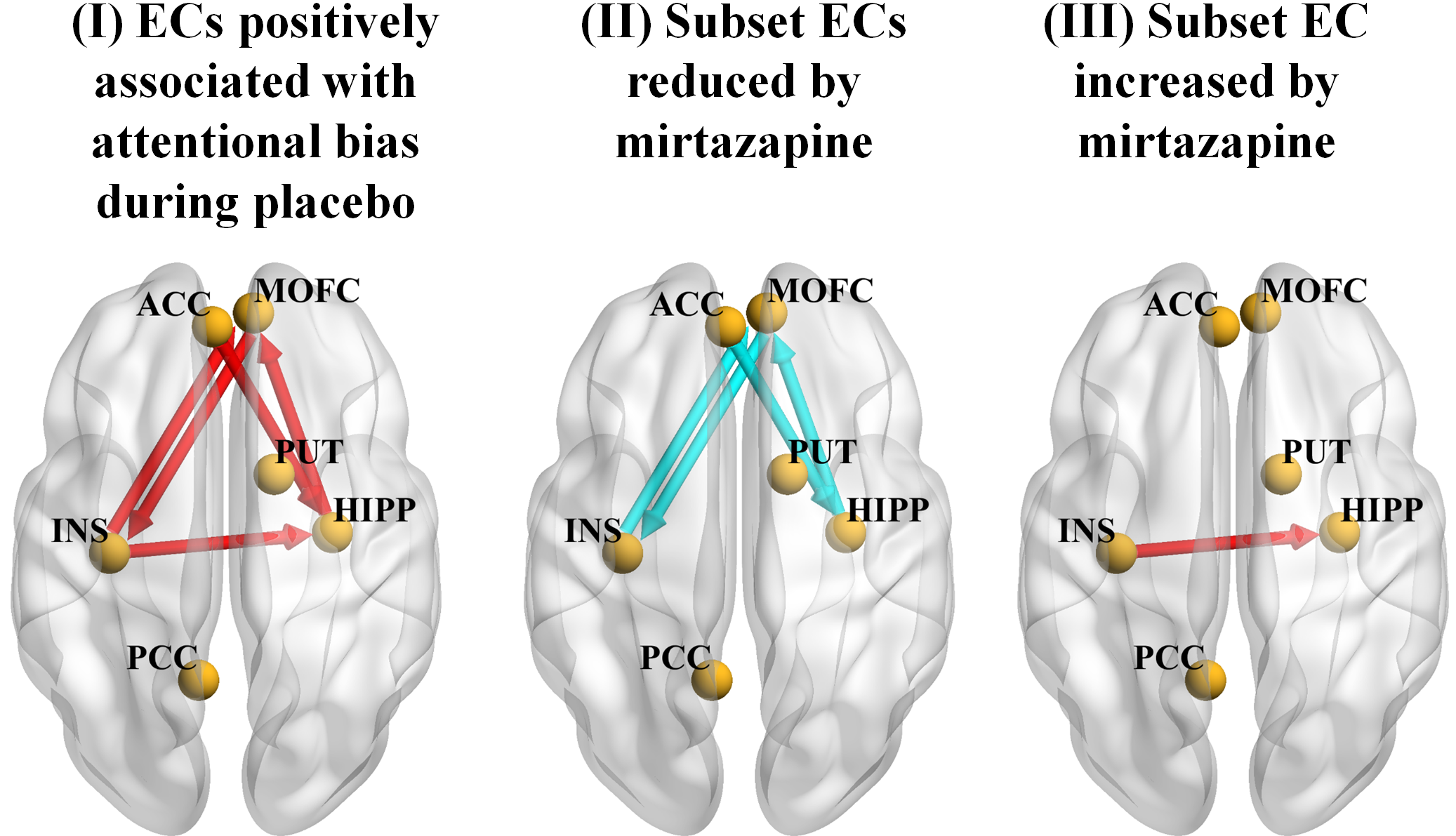
**

**SI-Figure 1:** The DCM results for all participants (n=28), visualized with the BrainNet Viewer (http://www.nitrc.org/projects/bnv/) [^12^](#_ENREF_56). The six DCM nodes are illustrated as **gold** spheres, and the lines with arrows represent the ECs. In the left panel (I), the ECs (including hypothesized L-ACC to R-HIPP EC) with placebo CW-modulation positively associated with the attentional bias are shown by **red** lines. In the middle panel (II), the four ECs (subset of those shown in the left panel, including hypothesized L-ACC to R-HIPP EC) reduced by the mirtazapine are shown by **light blue** lines. In the right panel (III), one EC (subset of those shown in the left panel) increased by the mirtazapine is shown by a **red** line. ACC, anterior cingulate cortex; MOFC, medial orbitofrontal cortex; INS, insula; HIPP, hippocampus; PCC, posterior cingulate cortex; PUT, putamen. The left side of this figure aligns with the left brain hemisphere.

**
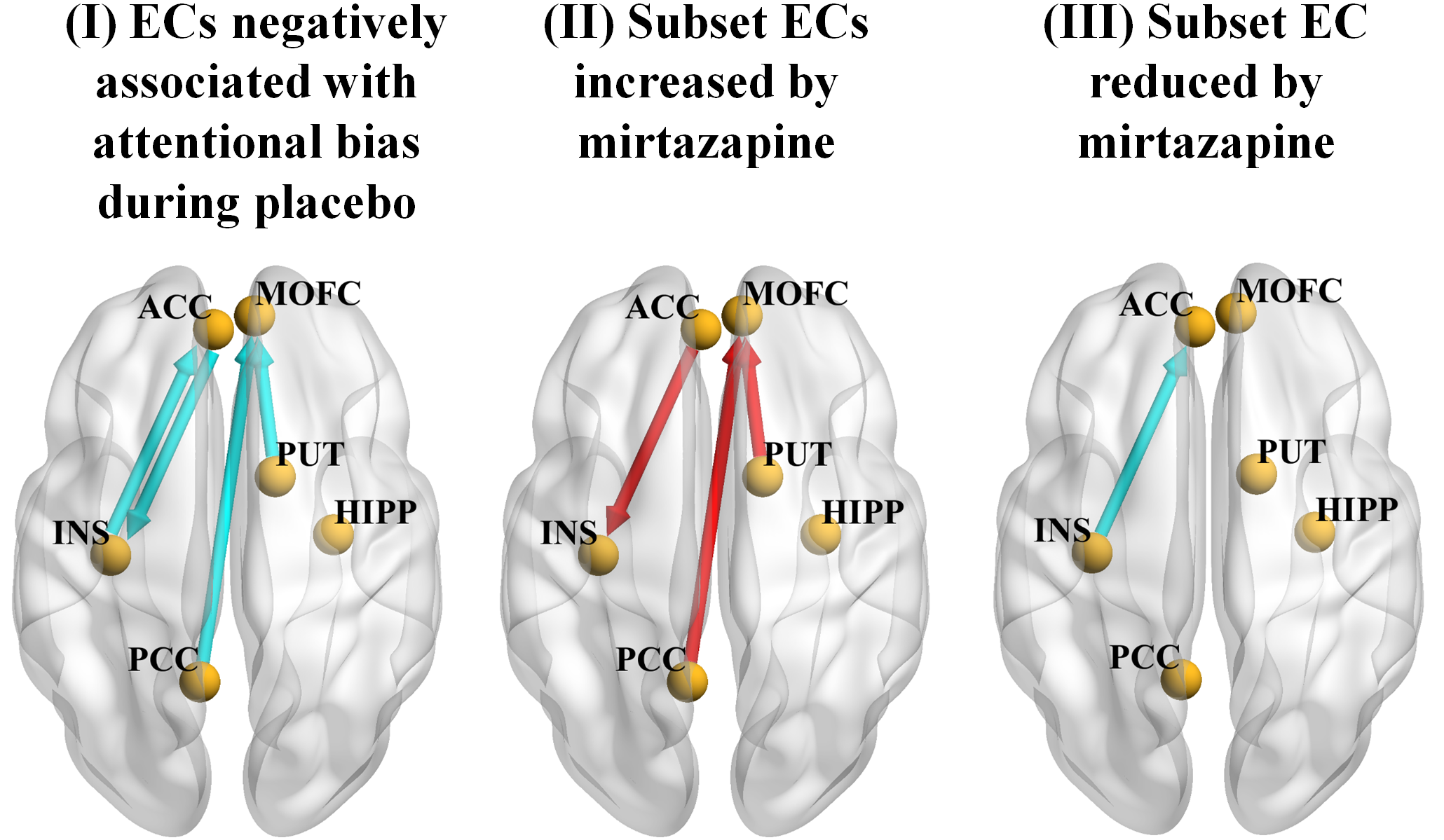
**

**SI-Figure 2:** Additional DCM results for all the participants (n=28), visualized with the BrainNet Viewer (http://www.nitrc.org/projects/bnv/) [^12^](#_ENREF_56). The six DCM nodes are illustrated as **gold** spheres, and the lines with arrows represent the ECs. In the left panel (I), the ECs with placebo CW-modulation negatively associated with the attentional bias are shown by **light blue** lines. In the middle panel (II), the three ECs (subset of those shown in the left panel) increased by the mirtazapine are shown by **red** lines. In the right panel (III), one EC (subset of those shown in the left panel) reduced by the mirtazapine is shown by a **light blue** line. ACC, anterior cingulate cortex; MOFC, medial orbitofrontal cortex; INS, insula; HIPP, hippocampus; PCC, posterior cingulate cortex; PUT, putamen. The left side of this figure aligns with the left brain hemisphere.

**SI-Table 1.** Several CUD participants (n=8) were excluded from those who initialized the experiment (n=36), with 28 participants included in the final analysis.

| **Reason for participant exclusion** | **Number of**  **Excluded Subject(s)** |
| --- | --- |
| Completion of only one scan | 2 |
| Positive urine screen for opioids | 1 |
| Unusable fMRI data (artifacts, incidental brain findings, undue head motions) | 3 |
| Female participants with C/G genotype | 2 |

**SI-Table 2.** The activation clusters (CW minus NW>0) found by the second level univariate SPM one-sample t-test analysis for the placebo scan and for all the participants, with cluster-defining threshold t=2.4 and uncorrected two-tailed cluster level p<0.05. These activation clusters (two brain regions highlighted by **bold** font) were used to constrain the spatial extent of two *a priori* DCM nodes (L-ACC and R-MOFC). Negative x = Left hemisphere. L=left. R=right.

| AAL2 Label | Number of voxels | Maximal t value within labeled region | MNI coordinates [x y z] (mm) of voxel with maximal t |
| --- | --- | --- | --- |
| L superior frontal gyrus | 132 | 3.02 | −22, 50, 2 |
| L middle frontal gyrus | 18 | 2.80 | −28, 52, −12 |
| R middle frontal gyrus | 69 | 3.78 | 42, 42, 18 |
| L opercularus part of inferior frontal gyrus | 102 | 4.70 | −40, 12, 14 |
| R triangularus part of inferior frontal gyrus | 45 | 3.75 | 36, 24, 14 |
| R orbitalus part of inferior frontal gyrus | 37 | 3.01 | 28, 26, −10 |
| L supplementary motor area | 33 | 3.59 | −8, 20, 62 |
| R supplementary motor area | 63 | 3.55 | 8, 6, 66 |
| R olfactory gyrus | 23 | 2.65 | 22, 8, −14 |
| L superior medial frontal cortex | 29 | 3.10 | −8, 56, 0 |
| L medial orbital frontal cortex | 37 | 3.76 | −12, 56, −2 |
| **R medial orbital frontal cortex** | **39** | **3.75** | **4, 51, −11** |
| R rectus gyrus | 10 | 2.43 | −8, 20, −12 |
| L insula | 19 | 2.81 | −38, −10, 2 |
| R insula | 106 | 4.37 | 32, 24, 14 |
| **L anterior cingulate cortex** | **87** | **3.57** | **−4, 47, 4** |
| R anterior cingulate cortex | 11 | 3.02 | 2, 44, 6 |
| R amygdala | 41 | 2.97 | 20, 0, −12 |
| L caudate | 51 | 3.19 | −12, 16, −6 |
| L putamen | 78 | 3.58 | −28, −4, −6 |
| L pallidum | 11 | 3.03 | −26, −6, −4 |

**SI-Table 3.** The activation clusters found by a two-sample t-test comparison of the two genotype groups (with cluster-defining threshold t=2.4 and uncorrected two-tailed cluster level p<0.05) showing that, during the mirtazapine scan, the participants with the wild-type *HTR2C* exhibited greater cocaine word-elicited activation than those with the *HTR2C* SNP. These activation clusters (four brain regions highlighted by **bold** font) were used to constrain the spatial extent of four *a priori* DCM nodes (L-PCC, L-insula, R-hippocampus, and R-putamen). Negative x = Left hemisphere. L=left. R=right.

| AAL2 Label | Number of voxels | Maximal t value within labeled region | MNI coordinates [x y z] (mm) of voxel with maximal t | |
| --- | --- | --- | --- | --- |
| Rrolandic operculum | 11 | 2.80 | 50, −4, 4 |  |
| **L insula** | **143** | **3.42** | **−34, −19, 5** |  |
| R insula | 81 | 2.98 | 44, −10, −4 |  |
| L middle cingulate cortex | 60 | 3.15 | −10, −42, 34 |  |
| R middle cingulate cortex | 51 | 4.32 | 8, 42, 32 |  |
| **L posterior cingulate cortex** | **45** | **3.59** | **−8, −56, 20** |  |
| L hippocampus | 69 | 2.91 | −32, −22, −16 |  |
| **R hippocampus** | **85** | **3.65** | **31, 13, −20** |  |
| L parahippocampal gyrus | 74 | 3.59 | −28, −36, −14 |  |
| L calcarine sulcus | 678 | 4.07 | 0, −66, −10 |  |
| R calcarine sulcus | 196 | 3.77 | 2, −64, 10 |  |
| L cuneus | 373 | 4.03 | 0, −80, 20 |  |
| R cuneus | 90 | 3.26 | 4, −78, 18 |  |
| L lingual gyrus | 114 | 3.75 | −2, −74, 6 |  |
| R lingual gyrus | 120 | 3.82 | 10, −58, 4 |  |
| L superior occipital gyrus | 100 | 3.51 | −16, −84, 12 |  |
| R superior occipital gyrus | 90 | 4.84 | 22, −78, 28 |  |
| L middle occipital gyrus | 92 | 3.49 | −12, −94, 0 |  |
| R middle occipital gyrus | 38 | 3.05 | 28, −76, 28 |  |
| L inferior occipital gyrus | 160 | 3.52 | −26, −90, −12 |  |
| L fusiform gyrus | 112 | 3.51 | −32, −76, −12 |  |
| L precuneus | 328 | 3.50 | 0, −58, 20 |  |
| R precuneus | 182 | 3.55 | 2, −58, 20 |  |
| R caudate | 59 | 3.30 | 8, 14, −8 |  |
| L putamen | 11 | 2.90 | −32, −18, −2 |  |
| **R putamen** | **109** | **3.29** | **20, 6, −6** |  |
| R pallidum | 14 | 3.20 | 16, 10, −4 |  |
| L thalamus | 11 | 2.64 | −6, −20, −2 |  |
| L superior temporal gyrus | 64 | 3.32 | −44, −22, 0 |  |
| R superior temporal gyrus | 68 | 3.36 | 54, −10, 0 |  |
| L middle temporal gyrus | 70 | 3.68 | −52, −16, −8 |  |
| L cerebellum | 27 | 2.73 | −4, −52, 0 |  |

**SI-Table 4.** The results of the DCM PEB analyses testing if the mean of placebo CW-modulation was different from zero, the linear relationship between the placebo CW-modulation on attentional bias, and if the mirtazapine-minus-placebo modulatory change was different from zero, for each EC across all the participants. An EC finding was considered reliable if Bayesian-PP>0.95 (corresponding to a Bayes-factor of 3). Bayesian posterior inference during the PEB analyses eschews the multiple comparison on ECs because of the lack of false positives (see main text for details). The hypothesized EC is highlighted by **bold** font. The eight non-hypothesized ECs related to the attentional bias and affected by the mirtazapine are highlighted by *italic* font. M = mean of the modulatory change (Hz), PP = Posterior Probability. *beta* = regression coefficient (slope) of the linear regression of EC on attentional bias. For example, a *beta* of 0.001 indicates a change in EC of 0.001 Hz per each ms change of attentional bias. ACC, anterior cingulate cortex; MOFC, medial orbital frontal cortex; PCC, posterior cingulate cortex; INS, insula; HIPP, hippocampus; PUT, putamen; L, left; and R, right.

| Effective Connectivity (EC) | Group mean of placebo CW-modulation across all participants (n=28) | | Linear regression analysis across all participants between CW-modulation and attentional bias (n=28) | | Group mean of mirtazapine-minus-placebo modulatory change, across all participants (n=28) | |
| --- | --- | --- | --- | --- | --- | --- |
|  | M | PP | *beta* | PP | M | PP |
| L-ACC to R-MOFC | 0.0944 | 1 | 0 | 0 | -0.6064 | 1 |
| L-ACC to L-PCC | -0.1400 | 1 | 0 | 0 | 0.2335 | 1 |
| *L-ACC to L-INS* | *-0.0867* | *1* | *-0.0041* | *1* | *0.4010* | *1* |
| **L-ACC to R-HIPP** | **-0.1218** | **1** | **0.0029** | **1** | **-0.2390** | **1** |
| L-ACC to R-PUT | 0 | 0 | 0 | 0 | -0.1928 | 1 |
| R-MOFC to L-ACC | 0.0681 | 1 | -0.0020 | 0.6500 | 0 | 1 |
| R-MOFC to L-PCC | 0.0562 | 0.7709 | 0 | 0 | -0.1588 | 1 |
| *R-MOFC to L-INS* | *0* | *0* | *0.0056* | *1* | *-0.2278* | *1* |
| R-MOFC to R-HIPP | 0.1614 | 1 | 0 | 0 | 0 | 0 |
| R-MOFC to R-PUT | -0.0988 | 1 | 0 | 0 | 0 | 0 |
| L-PCC to L-ACC | -0.0982 | 1 | 0 | 0 | 0.2978 | 1 |
| *L-PCC to R-MOFC* | *-0.1356* | *1* | *-0.0047* | *1* | *0.2380* | *1* |
| L-PCC to L-INS | 0.1719 | 1 | 0 | 0 | 0 | 0 |
| L-PCC to R-HIPP | -0.0511 | 0.8014 | -0.0028 | 0.7685 | -0.0333 | 0.4131 |
| L-PCC to R-PUT | -0.0934 | 1 | 0.0021 | 0.6178 | 0 | 0 |
| *L-INS to L-ACC* | *0.0921* | *1* | *-0.0027* | *1* | *-0.2140* | *1* |
| *L-INS to R-MOFC* | *0.1547* | *1* | *0.0045* | *1* | *-0.1528* | *1* |
| L-INS to L-PCC | 0.0686 | 1 | 0 | 0 | 0 | 0 |
| *L-INS to R-HIPP* | *0.1759* | *1* | *0.0070* | *1* | *0.1611* | *1* |
| L-INS to R-PUT | 0.1167 | 1 | -0.0031 | 1 | 0 | 0 |
| R-HIPP to L-ACC | -0.1354 | 1 | 0 | 0 | -0.2026 | 1 |
| *R-HIPP to R-MOFC* | *0.1463* | *1* | *0.0063* | *1* | *-0.2060* | *1* |
| R-HIPP to L-PCC | 0 | 0 | 0 | 0 | 0 | 0 |
| R-HIPP to L-INS | 0.1055 | 1 | 0 | 0 | 0 | 0 |
| R-HIPP to R-PUT | 0.1664 | 1 | 0 | 0 | -0.1101 | 0.9943 |
| R-PUT to L-ACC | 0 | 0 | -0.0033 | 1 | 0 | 0 |
| *R-PUT to R-MOFC* | *-0.1482* | *1* | *-0.0034* | *1* | *0.2164* | *1* |
| R-PUT to L-PCC | 0 | 0 | 0 | 0 | 0 | 0 |
| R-PUT to L-INS | 0.0790 | 0.8308 | 0 | 0 | 0 | 0 |
| R-PUT to R-HIPP | 0.1204 | 1 | -0.0061 | 1 | 0 | 0 |

**SI-Table 5.** The results of the DCM PEB analyses comparing the ECs between the participants with wild-type *HTR2C*(n=15) and *HTR2C* SNP(n=13) during placebo scan and for the effects of the mirtazapine. An EC finding was considered reliable if Bayesian-PP>0.95 (corresponding to a Bayes-factor of 3). Bayesian posterior inference during the PEB analyses eschews the multiple comparison on ECs because of the lack of false positives (see main text for details). The hypothesized EC is highlighted by **bold** font. M = mean of the modulatory change (Hz), PP = Posterior Probability. ACC, anterior cingulate cortex; MOFC, medial orbital frontal cortex; PCC, posterior cingulate cortex; INS, insula; HIPP, hippocampus; PUT, putamen; L, left; and R, right.

| Effective Connectivity (EC) | Subgroup difference (*HTR2C* SNP minus wild-type *HTR2C*) in placebo CW-modulation | | Subgroup difference in mirtazapine-minus-placebo modulatory change (*HTR2C* SNP minus wild-type *HTR2C*) | |
| --- | --- | --- | --- | --- |
|  | M | PP | M | PP |
| L-ACC to R-MOFC | 0 | 0 | 0 | 0 |
| L-ACC to L-PCC | -0.2294 | 1 | 0.4721 | 1 |
| L-ACC to L-INS | -0.0959 | 1 | 0.1430 | 1 |
| **L-ACC to R-HIPP** | **0** | **0** | **0.7533** | **1** |
| L-ACC to R-PUT | -0.1008 | 1 | 0.3517 | 1 |
| R-MOFC to L-ACC | 0 | 0 | -0.2132 | 1 |
| R-MOFC to L-PCC | 0 | 0 | -0.1029 | 1 |
| R-MOFC to L-INS | 0 | 0 | 0 | 0 |
| R-MOFC to R-HIPP | 0 | 0 | -0.1696 | 1 |
| R-MOFC to R-PUT | 0 | 0 | -0.0990 | 1 |
| L-PCC to L-ACC | 0 | 0 | 0.1947 | 1 |
| L-PCC to R-MOFC | 0.2151 | 1 | 0.0398 | 0.4597 |
| L-PCC to L-INS | 0.1575 | 1 | -0.1942 | 1 |
| L-PCC to R-HIPP | 0 | 0 | -0.1861 | 1 |
| L-PCC to R-PUT | 0.3845 | 1 | -0.1284 | 1 |
| L-INS to L-ACC | -0.2336 | 1 | -0.3141 | 1 |
| L-INS to R-MOFC | -0.3427 | 1 | -0.1202 | 1 |
| L-INS to L-PCC | 0 | 0 | 0 | 0 |
| L-INS to R-HIPP | 0.2056 | 1 | 0 | 0 |
| L-INS to R-PUT | -0.3633 | 1 | 0 | 0 |
| R-HIPP to L-ACC | 0.1240 | 1 | 0 | 0 |
| R-HIPP to R-MOFC | 0.4066 | 1 | -0.0987 | 0.9022 |
| R-HIPP to L-PCC | 0.1035 | 1 | 0 | 0 |
| R-HIPP to L-INS | 0.1108 | 1 | 0 | 0 |
| R-HIPP to R-PUT | -0.1562 | 1 | 0 | 0 |
| R-PUT to L-ACC | -0.0675 | 0.7519 | 0.6238 | 1 |
| R-PUT to R-MOFC | 0 | 0 | 0.2710 | 1 |
| R-PUT to L-PCC | 0.2301 | 1 | -0.2300 | 1 |
| R-PUT to L-INS | 0 | 0 | -0.1293 | 0.9809 |
| R-PUT to R-HIPP | -0.1392 | 1 | -0.3369 | 1 |

**References**

1. Ma L, Steinberg JL, Cunningham KA, Bjork JM, Lane SD, Schmitz JM *et al.* Altered anterior cingulate cortex to hippocampus effective connectivity in response to drug cues in men with cocaine use disorder. *Psychiatry Res Neuroimaging* 2018; **271:** 59-66.

2. Cox RW. AFNI: software for analysis and visualization of functional magnetic resonance neuroimages. *Comput Biomed Res* 1996; **29**(3)**:** 162-173.

3. Friston KJ. Statistical parametric maps in functional imaging: a general linear approach. *Human Brain Mapping* 1995; **2:** 189-210.

4. Holmes AP, Friston KJ. Generalisability, random effects and population inference. *NeuroImage* 1998; **7:** S754.

5. Friston KJ, Harrison L, Penny W. Dynamic causal modelling. *Neuroimage* 2003; **19**(4)**:** 1273-1302. PMID: 12948688.

6. Rolls ET, Huang CC, Lin CP, Feng J, Joliot M. Automated anatomical labelling atlas 3. *Neuroimage* 2020; **206:** 116189.

7. Friston KJ, Penny W. Posterior probability maps and SPMs. *Neuroimage* 2003; **19**(3)**:** 1240-1249.

8. Van Overwalle F, Van de Steen F, Marien P. Dynamic causal modeling of the effective connectivity between the cerebrum and cerebellum in social mentalizing across five studies. *Cogn Affect Behav Neurosci* 2019; **19**(1)**:** 211-223.

9. Ipek. Normality test package (https://www.mathworks.com/matlabcentral/fileexchange/60147-normality-test-package), MATLAB CentralFile Exchange. Retrieved September 19, 2020. 2020.

10. Friston KJ, Li B, Daunizeau J, Stephan KE. Network discovery with DCM. *Neuroimage* 2011; **56**(3)**:** 1202-1221. PMID: 21182971.

11. Volkow ND, Fowler JS. Addiction, a disease of compulsion and drive: involvement of the orbitofrontal cortex. *Cereb Cortex* 2000; **10**(3)**:** 318-325.

12. Xia M, Wang J, He Y. BrainNet Viewer: a network visualization tool for human brain connectomics. *PLoS One* 2013; **8**(7)**:** e68910.
